# Supplementary material for: Trade-offs shaping transmission of sylvatic dengue and Zika viruses in monkey hosts
Source: Nat Commun. 2024 Mar 27;15:2682. doi: 10.1038/s41467-024-46810-x (PMC10973334; doi:10.1038/s41467-024-46810-x)
Supplement: Supplementary file 1 — Supplementary Information [file 41467_2024_46810_MOESM1_ESM.pdf]

Supplementary Information

Trade-offs shaping transmission of sylvatic dengue and Zika virus in native and novel monkey hosts

Kathryn A. Hanley, Hélène Cecilia, Sasha R. Azar, Brett Moehn, Jordan Gass, Natalia I. Oliveira da Silva, Wanqin Yu, Ruimei Yun, Benjamin M. Althouse, Nikos Vasilakis, Shannan L. Rossi

Note : Supplementary Data 1-6 are provided as separate files.

Temperature and weight of non-human primates over the course of the experiment

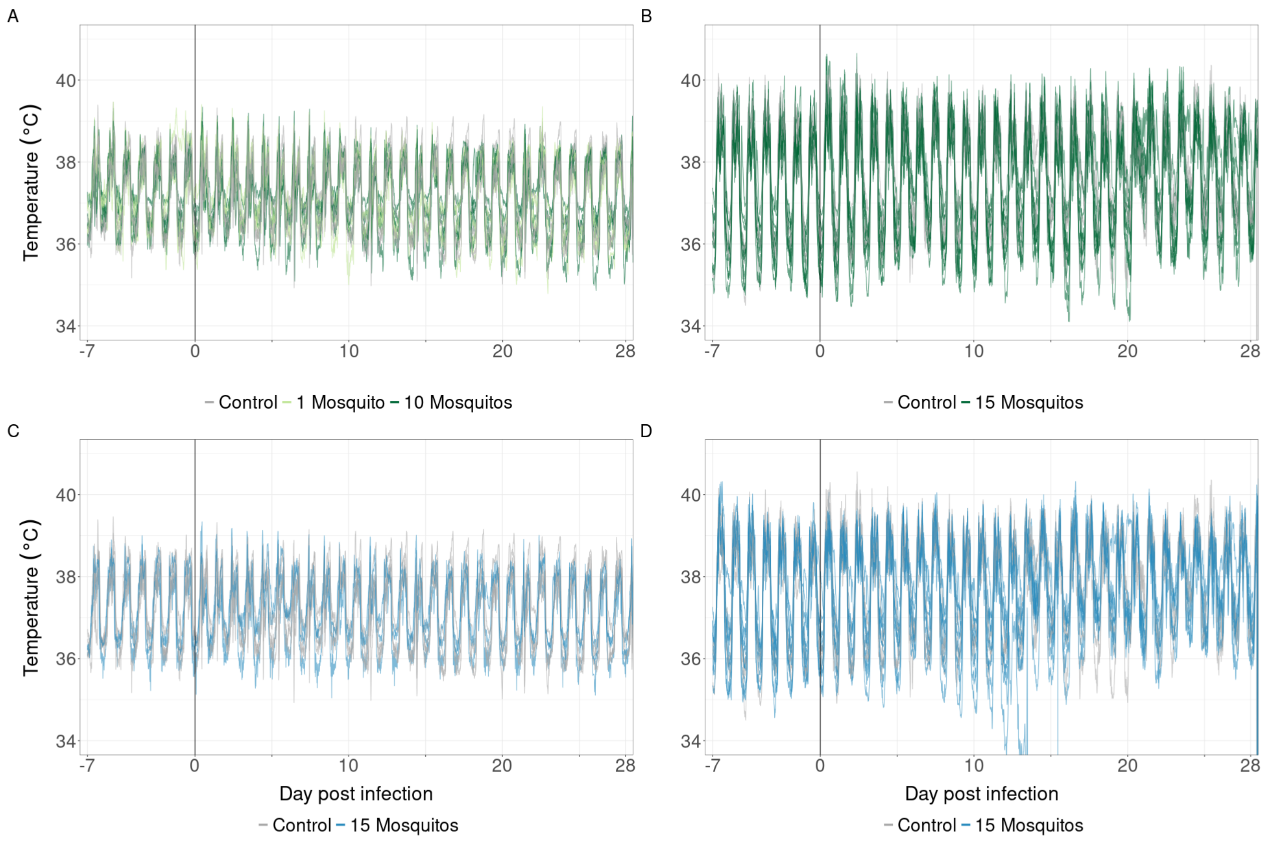

Figure S.1: Changes in temperature in cynomolgus macaques (A, C) and squirrel monkeys (B, D), infected with DENV (green lines, A, B) or ZIKV (blue lines, C, D) or control (grey lines, A-D). Note that the same set of control animals are shown for same species experiments (left and right columns). The two animals in panel D that drop below 34°C were the two animals that were euthanized before the end of the experiment. Normal temperature ranges are [36;39.5]°C for cynomolgus macaques[1,2] and [33.3;41]°C for squirrel monkeys[3,4]. Source data are provided as a Source Data file.

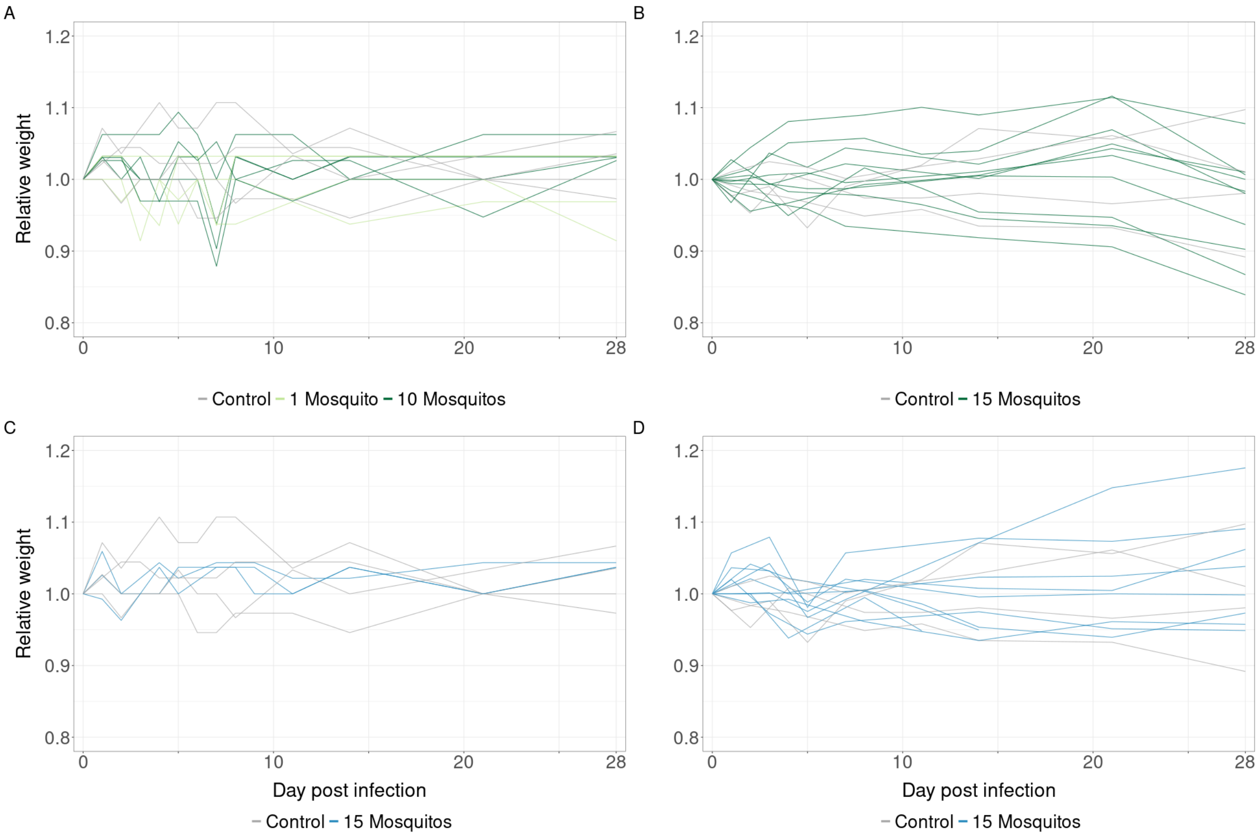

Figure S.2: Changes in body weight in cynomolgus macaques (A, C) and squirrel monkeys (B, D), infected with DENV (green lines, A, B) or ZIKV (blue lines, C, D) or control (grey lines, A-D). Note that the same set of control animals are shown for same species experiments (left and right columns). Source data are provided as a Source Data file.

S.1 Differences in dose delivered to NHPs on day 0

The number of infectious bites initially received per NHP, as well as the viral titers contained in biting mosquitoes' saliva, are the components driving the initial dose delivered to NHPs, which can then drive their viral dynamics and immune response. Differences between experiments regarding those factors were assessed either descriptively or using linear models (Sections S.1.1-S.1.3).

S.1.1 Number of infectious bites

The proportion of positive bites was computed based on mosquitoes that fed and survived to titer. We applied this proportion to the number of mosquitoes that fed, and rounded to the nearest integer, to estimate the true number of positive bites each NHP got, which was our response variable. This added an additional positive bite in 5/28 cases. Cynomolgus macaques from the low exposure group all received 1 DENV-infectious bites so we don't mention them here.

As can be seen in the table below, the number of DENV-infectious bites received per NHP was on average lower than the number of ZIKV-infectious bites received per NHP. For a given virus, the number of infectious bites received per NHP was similar in squirrel monkeys and cynomolgus macaques.

Virus	NHP species	Number of infectious bites per NHP		
		Mean	Min	Max
DENV	Squirrel monkeys	3.4	2	6
DENV	Cynomolgus macaques (high exposure)	4.2	3	6
ZIKV	Squirrel monkeys	6	1	11
ZIKV	Cynomolgus macaques	7.3	5	10

S.1.2 Saliva titer per mosquito

A Levene's test was conducted and showed a lower variability of $\log_{10}$ saliva titers in DENV- than ZIKV-infected mosquitoes (F = 12.7, p = 5e-4). For this test, a random value between 1 and LOD PFU was assigned to samples positive only after passage in C6/36 cells, which were overrepresented in the DENV group. This was therefore a conservative choice, likely to overestimate the variance in this group.
We then used a linear model to detect differences in average $\log_{10}$ saliva titers between DENV- and ZIKV-infected mosquitoes. To account for the heteroskedasticity detected by the Levene's test, we added a dispersion model. The saliva titers of ZIKV-infected *Ae. albopictus* were significantly higher than those of DENV-infected *Ae. albopictus* (ZIKV 2.55 [2.36 ; 2.74] $\log_{10}$ PFU, DENV 1.38 [1.24 ; 1.53], p = 2.2e-4).

```
 Family: gaussian  ( identity )
Formula:          log_titer ~ virus
Dispersion:                 ~virus
Data: df_randomLOD

     AIC      BIC   logLik deviance df.resid
   305.8    317.4   -148.9    297.8      132


Conditional model:
                Estimate Std. Error z value Pr(>|z|)
(Intercept)      1.38493    0.07395  18.727   <2e-16 ***
virusZika virus  1.16145    0.12237   9.491   <2e-16 ***
---
Signif. codes:  0 '***' 0.001 '**' 0.01 '*' 0.05 '.' 0.1 ' ' 1

Dispersion model:
                Estimate Std. Error z value Pr(>|z|)
(Intercept)      -1.1833     0.1890  -6.262 3.81e-10 ***
virusZika virus   0.9096     0.2464   3.691 0.000223 ***
---
Signif. codes:  0 '***' 0.001 '**' 0.01 '*' 0.05 '.' 0.1 ' ' 1
```

S.1.3 Dose delivered to NHPs

We summed saliva titers of mosquitoes biting a same NHP to estimate the dose delivered. For those positive only after passage in C6/36 cells, a value of half the limit of detection was assigned (20 or 5 PFU depending on experiments).

As can be seen in the table below, DENV doses received by squirrel monkeys and cynomolgus macaques from the high exposure group were on average similar. Cynomolgus macaques from the low exposure group received lower DENV doses than those from the high exposure group. Lower doses of ZIKV were received by squirrel monkeys compared to cynomolgus macaques. Lower doses of DENV than ZIKV were received by both squirrel monkeys and cynomolgus macaques.

**Virus**	**NHP species**	**Estimated dose delivered per NHP ($\log_{10}$ PFU)**		
		Mean	Min	Max
DENV	Squirrel monkeys	2.65	1.60	3.27
DENV	Cynomolgus macaques (high exposure)	1.92	1.78	2.08
DENV	Cynomolgus macaques (low exposure)	1.30	1.30	1.30
ZIKV	Squirrel monkeys	3.68	2.81	4.20
ZIKV	Cynomolgus macaques	4.44	4.41	4.48

S.2 Individual kinetics of ZIKV replication and transmission

Two monkeys infected with ZIKV, 4683 and 4728, had to be euthanized prior to the end of the experiment following the recommendation of the head veterinarian on staff. On Day 13, NHP 4683 was found during morning rounds hypothermic and hypoglycemic at the bottom of the cage. Shortly after, cluster seizures were observed, and euthanasia was elected. A lesion in the occipital-temporal lobe likely led to the seizures resulting the hypothermia and hypoglycemia. No samples were taken at necropsy to analyze for the presence of ZIKV. Clinical presentations were inconsistent with previous ZIKV reports, but infection causation cannot be ruled out. On Day 15, NHP 4728 had a worsening sore on the back left foot heel that failed to respond for more than 2 weeks to repeated treatments of chlorohexidine and silver sulfadine. These two animals had the lowest weight of all 12 female squirrel monkeys prior to infection; while the mean weight of female squirrel monkeys in this experiment was 676.1g ± 21g, NHPs 4728 and 4683 weighed 555g and 583g, respectively. These two NHPs also produced the two highest titers of ZIKV of all 10 monkeys infected with the virus (Figure S.3).

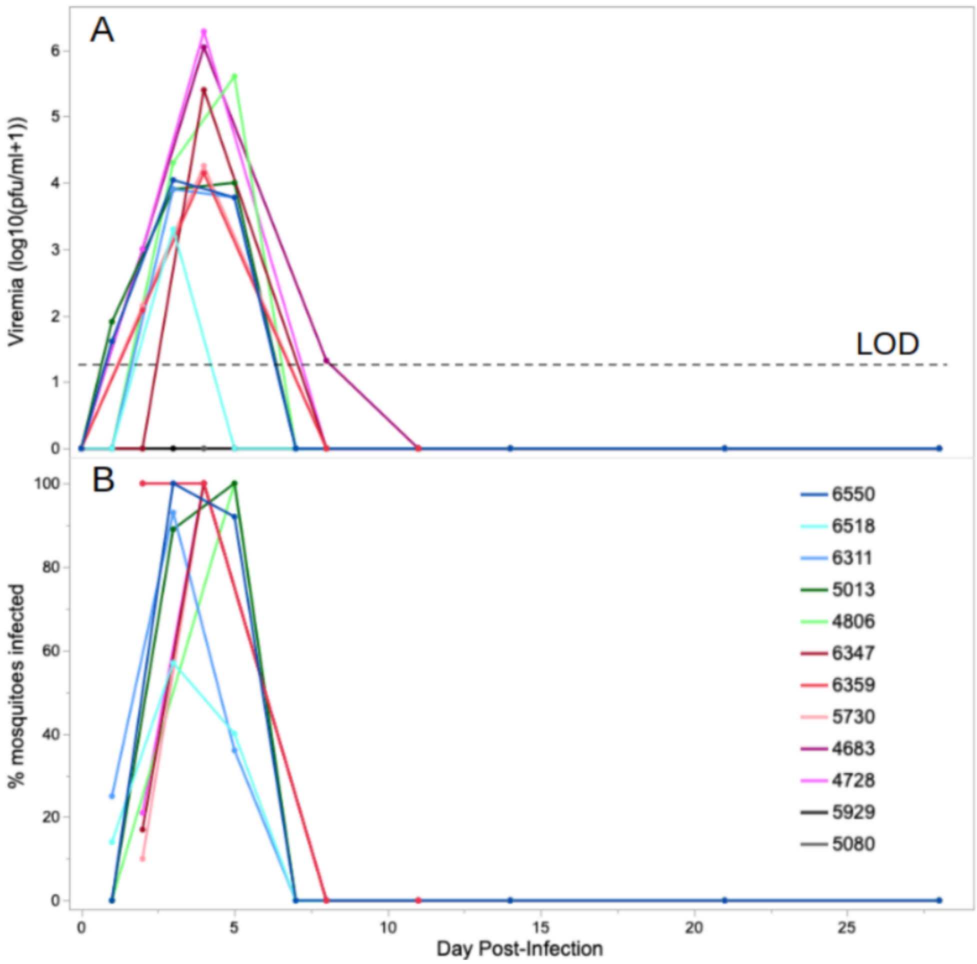

Figure S.3: ZIKV viremia (A) and percentage of mosquitoes infected (B) at designated day post-infection for designated squirrel monkey. Dark lines indicate control animals, blue and purple tone lines indicate cohort 1, and red and orange tone lines indicate cohort 2. Horizontal dashed line shows the limit of detection. Source data are provided as a Source Data file.

For analyses performed in Sections S.3 and S.4, we corrected some of our initial data to be able to distinguish between likely true absence of viremia and undetectable levels, for the fitting of dose-response relationships. In some cases, non-human primates (NHPs) had no detectable viremia, even after one passage in Vero cells, but did show transmission to mosquitoes (body and/or leg). We assigned a viremia of 10 PFU/ml (half the limit of detection) to these cases. Similarly, if a NHP was not detectably viremic nor transmitted to mosquitoes on a given sampling day, but was viremic and/or transmitted to mosquitoes on the previous and following sampling days, we also assigned a viremia of 10 PFU/ml. Lastly, a cynomolgus macaques which transmitted virus to mosquitoes on day 8 was assigned a viremia of 10 PFU/ml, in the absence of actual viremia measurement. The remaining samples with no detectable viremia even after passage, and no transmission to mosquitoes were considered as true absence of viremia. The resulting data is presented in Figure S.5, and is refered as deduced viremia.

S.3 Comparison of Dengue and Zika virus transmission from squirrel monkeys to *Aedes albopictus*

To quantify a possible relationship between host infectious titer ($\log_{10}$ PFU/ml) and probability to infect mosquitoes, we first used a flexible fitting approach known as generalized additive model. The probability of mosquito infection was broadly defined, measured by a positive mosquito body or leg. We used a binomial error distribution, and constrained the number of knots (i.e the number of polynomials composing the final curve) to 6. Separate relationships were fitted for dengue and Zika viruses, and transmission from both NHP species was considered for dengue. See Figure 5 in main text.

S.3.1 Dengue virus transmission

```
Family: binomial
Link function: logit

Formula:
  cbind(k, N - k) ~ s(log_V, k = 6)

Parametric coefficients:
  Estimate Std. Error z value Pr(>|z|)
(Intercept)   -17.43      37.04  -0.471    0.638

Approximate significance of smooth terms:
  edf Ref.df Chi.sq p-value
s(log_V) 1.841  1.995  4.173   0.118

R-sq.(adj) =  0.409   Deviance explained = 67.4%
UBRE = -0.47875  Scale est. = 1         n = 157
```

S.3.2 Zika virus transmission

```
Family: binomial
Link function: logit

Formula:
  cbind(k, N - k) ~ s(log_V, k = 6)

Parametric coefficients:
  Estimate Std. Error z value Pr(>|z|)
(Intercept)  -2.9402     0.8104  -3.628 0.000285 ***
  ---
  Signif. codes:  0 ‘***’ 0.001 ‘**’ 0.01 ‘*’ 0.05 ‘.’ 0.1 ‘ ’ 1

Approximate significance of smooth terms:
  edf Ref.df Chi.sq p-value
```

```
s(log_V) 3.232  3.611  68.25  <2e-16 ***
  ---
  Signif. codes:  0 '***' 0.001 '**' 0.01 '*' 0.05 '.' 0.1 ' ' 1

R-sq.(adj) =  0.844   Deviance explained = 85.2%
UBRE = 0.14002  Scale est. = 1         n = 64
```

S.4 Comparison of Zika Virus Transmission From Squirrel Monkeys and Dengue Virus Transmission From Humans

We fitted three different functional forms (Eqs. S.1-S.3) to the data, using a maximum likelihood approach. In these equations, p stands for the probability to infect a vector and V is the infectious titer of the host, on a linear scale. Each functional form was fitted with either a binomial likelihood or a beta-binomial likelihood, the latter accounting for overdispersion in the data. We used AICc to select the functional form providing the best fit to data[7]. This fitting procedure was applied twice to obtain separate relationships for mosquitoes' body and leg infection. To not constrain the fitting at the origin, we excluded data considered as true absence of viremia with no transmission to mosquitoes.

$$Logistic: p(V) = \frac{1}{1+\exp(-\beta_1(\log_{10}(V) - \log_{10}(\beta_0)))} \tag{S.1}$$

$$Ferguson: p(V) = 1 - \exp(-(\frac{\log_{10}(V)}{\theta_0})^{\theta_1}) \tag{S.2}$$

$$Hill: p(V) = \frac{\log_{10}(V)^{\gamma_1}}{\gamma_0 + \log_{10}(V)^{\gamma_1}} \tag{S.3}$$

Eq. S.1 is the logistic function, with the curve's maximum value fixed at 1 to be on the scale of probabilities. Eq. S.2 [8] was used to study vector competence for dengue when carrying Wolbachia. Eq. S.3, also called the Hill equation, is often used to model biological interactions that demonstrate sigmoidal response, in particular to capture the biomolecular interaction exhibiting cooperativity among two binding molecules[9]. To visualize the uncertainty around fitted curves, we sampled 5000 parameter sets using multivariate normal sampling with the covariance matrix of the fitting procedure.

In the study by Nguyen et al. 2013[10], data came from patients hospitalized at Ho-Chi-Minh city hospital in Vietnam between April and December 2011. DENV-1 cases were associated with the Genotype 1 lineage (different clades), and DENV-2 cases mostly with the Asian 1 lineage (a few Cosmopolitan). Cases with serotypes 3 and 4 were not sequenced. Mosquito infection was measured through the presence of virus in mosquito abdomens. The data we retrieved from the supplementary material of ten Bosch et al. 2018[11] contained 105 data points (28 DENV-1, 13 DENV-2, 16 DENV-3, 48 DENV-4), which is clearly only a subset of the data presented in Figure 2 of [10] (more than 260 data points estimated visually). Note that we did not get an answer from the senior author of [10] when emailed, and that we could not find a valid email address for the first author of the paper.

In the study by Duong et al. 2015[12], viral load and transmission data came from Cambodian participants (Kampong Cham province) infected between June and October of 2012 and 2013. DENV-1 cases were associated with the Genotype 1 lineage, DENV-2 cases with the Asian 1 lineage, and DENV-3 cases with Genotype 1 lineage (we excluded the two DENV-3 datapoints as it was insufficient for fitting). Mosquito infection was measured through the presence of virus in legs and wings. We did not distinguish between classes of disease severity (symptomatic, pre-symptomatic, asymptomatic) in our analyses.

In the study by Long et al. 2019[13], data came from a febrile surveillance study, along with a contact cluster study, in the Amazonian city of Iquitos, Peru. All infections were with Asian/American DENV-2 genotype. We retrieved data from direct mosquito feeding only. Mosquito infection was measured through the presence of virus in mosquito bodies, and heads, by conventional RT-PCR. We used the latter as a marker of disseminated infection, to compare with our data on mosquito legs.

The conversion factors used to transform RNA-emia data into infectious titers were retrieved from Blaney et al. 2005[14], which used strains DENV-1 Nauru/74, DENV-2 Tonga/74, DENV-3 Sleman/78, and DENV-4 Dominica/81. The differ-

ence between $\log_{10}$ genome equivalents/ml of serum and $\log_{10}$ PFU/ml of serum were 1.9 for DENV-1, 2.8 for DENV-2, 2.5 for DENV-3, and 1.9 for DENV-4. Because of this conversion, the curve fitted to the DENV-2 dataset from [12] was quite different from the one showed in the initial paper. Indeed, most points were shifted to the left, except for 2 points which corresponded to an absence of detectable viremia with transmission to mosquitoes. Those could not be excluded but were now closer to other points than in the initial RNA-emia scale. All fitting was done on $\log_{10}$(viremia + 1) to accommodate values below 1 PFU/ml. This conversion was not applied to data from Long et al.[13] as viral loads were already expressed in FFU/ml. However, our fits differ from those showed in the Long et al. paper because we included points with undetectable viremia and transmission to mosquitoes, and we accounted for overdispersion in the fitting.

Tables S.1-S.3 present the results of model selection and the resulting parameter estimates, for Zika virus only. Figure S.6 presents all DENV dose-response curves relationship fitted along with the data used. Results regarding dose-response relationships using presence of virus in mosquito legs are presented in the main text.
The best dose-response fit to characterize the relationship between host viremia and disseminated ZIKV infection in mosquitoes (presence of virus in mosquito legs) was obtained using a logistic equation (Eq. S.1) and a betabinomial likelihood, for both cynomolgus macaques, squirrel monkeys, and both species considered at once (Table S.1). This was also the case for DENV-1 data from Duong et al.[12], and DENV-2 data from Long et al.[13]. For DENV-4 data from Duong et al.[12], the best fit was obtained using the Hill equation (Eq. S.3) and a betabinomial likelihood.

The best dose-response fit to characterize the relationship between viremia and transmission (measured as infection of the mosquito bodies) of Zika virus by squirrel monkeys was obtained using a logistic equation (Eq. S.1) and a betabinomial likelihood, whereas for cynomolgus macaques it was using Eq. S.2 with a binomial likelihood (Table S.2). When both species were considered at once, the best fit was obtained using a logistic equation (Eq. S.1) and a betabinomial likelihood. For data from Nguyen et al. [10] and Long et al.[13], the best fit was using a logistic equation (Eq. S.1) and a betabinomial likelihood, for all serotypes (Figure S.6). To compare these curves, we report the estimations of the dose needed to infect the bodies of 50% of mosquitoes in Figure S.4.

**Dose-response functional form**	**Likelihood**	**Number of parameters**	**AICc (leg)**		
			**C**	**S**	**C+S**
Logistic (Eq. S.1)	binomial	2	47.3	101.7	158.3
	beta-binomial	3	**46.7**	**77.5**	**121.1**
Ferguson (Eq. S.2)	binomial	2	48.2	105.5	164.4
	beta-binomial	3	46.9	78.9	123.6
Hill (Eq. S.3)	binomial	2	54.4	113.6	175.2
	beta-binomial	3	49.9	81.6	126.8

Table S.1: Model selection for the dose-response relationships predicting the probability of *Aedes albopictus* leg infection based on infectious titers of cynomolgus macaques (C), squirrel monkeys (S) or both (C+S). The models with lowest corrected Akaike Information Criterion (AICc, highlighted in bold) are selected. When models using a same functional form, with binomial and betabinomial likelihood, were within 2 AICc difference, we checked with a likelihood ratio test which one to select.

**Dose-response functional form**	**Likelihood**	**Number of parameters**	**AICc (body)**		
			**C**	**S**	**C+S**
Logistic (Eq. S.1)	binomial	2	37.6	82.3	135.3
	beta-binomial	3	41.5	**79.6**	**124.0**
Ferguson (Eq. S.2)	binomial	2	**36.7**	82.6	139.9
	beta-binomial	3	40.9	80.5	127.0
Hill (Eq. S.3)	binomial	2	40.8	82.5	139.6
	beta-binomial	3	44.4	80.9	128.0

Table S.2: Model selection for the dose-response relationships predicting the probability of *Aedes albopictus* body infection based on infectious titers of cynomolgus macaques (C), squirrel monkeys (S) or both (C+S). The models with lowest corrected Akaike Information Criterion (AICc, highlighted in bold) are selected. When models using a same functional form, with binomial and betabinomial likelihood, were within 2 AICc difference, we checked with a likelihood ratio test which one to select.

NHP species	Vector body infection			Vector leg infection		
	**Parameter**	**Point-estimate**	**95% CI**	**Parameter**	**Point-estimate**	**95% CI**
C	θ_0	4.17	[3.93 ; 4.43]	$\log_{10}(\beta_0)$	3.84	[3.35 ; 4.32]
	θ_1	5.23	[3.23 ; 7.23]	β_1	1.59	[0.54 ; 2.64]
	overdispersion	NA	NA	overdispersion	9.08	[-6.10 ; 24.27]
S	$\log_{10}(\beta_0)$	5.00	[4.09 ; 5.93]	$\log_{10}(\beta_0)$	2.53	[1.96 ; 3.13]
	β_1	0.70	[0.36 ; 1.04]	β_1	1.12	[0.59 ; 1.64]
	overdispersion	8.58	[-3.36 ; 20.53]	overdispersion	2.31	[-0.02 ; 4.65]
C+S	$\log_{10}(\beta_0)$	4.16	[3.71 ; 4.60]	$\log_{10}(\beta_0)$	2.86	[2.32 ; 3.41]
	β_1	0.96	[0.57 ; 1.35]	β_1	0.94	[0.56 ; 1.33]
	overdispersion	5.20	[0.02 ; 10.38]	overdispersion	2.64	[0.57 ; 4.71]

Table S.3: Parameter estimates for the selected models of Zika virus transmission from cynomolgus macaques (C), squirrel monkeys (S), or both (C+S) to *Aedes albopictus*.

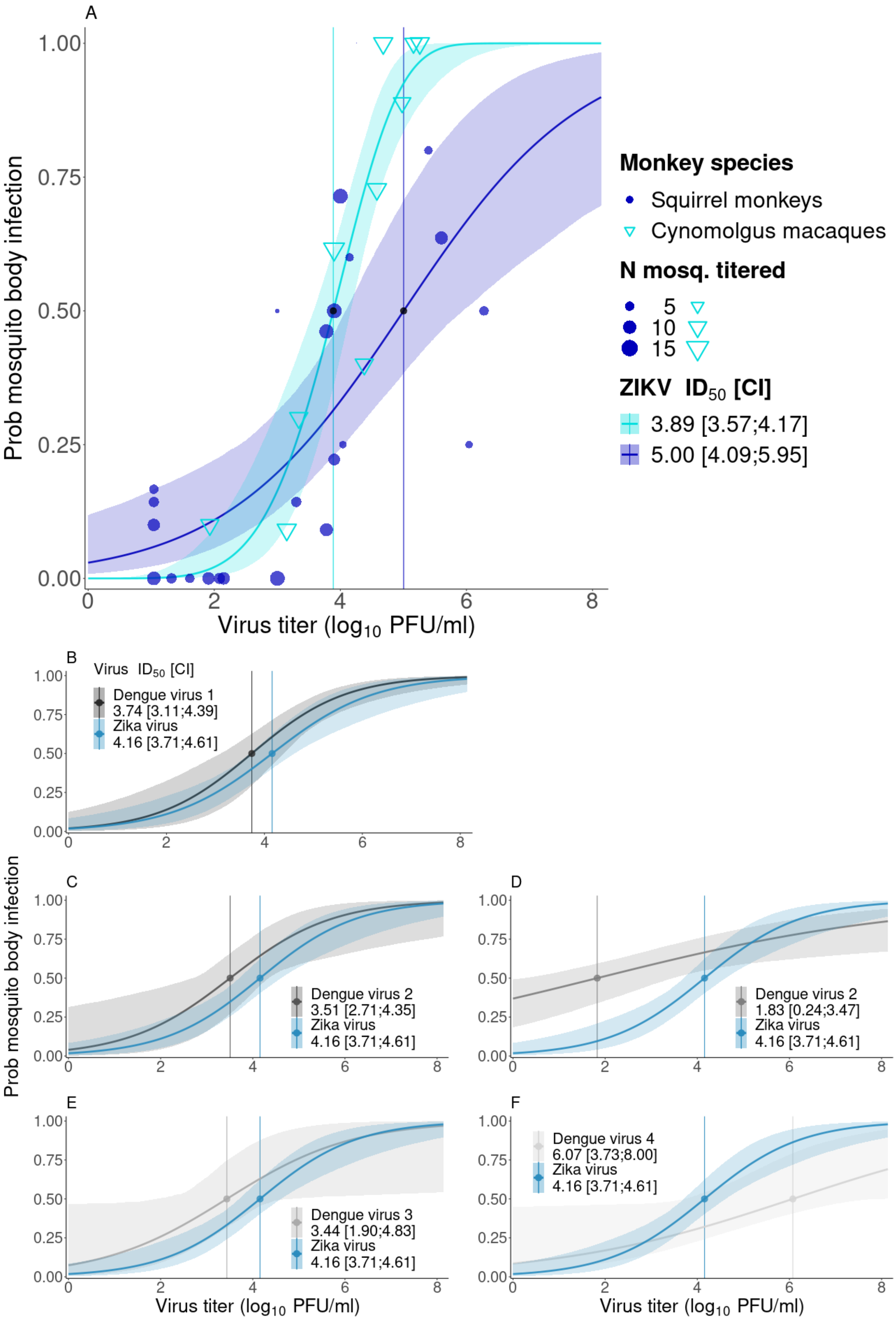

Figure S.4: (Caption next page)

Figure S.4: (Previous page.) A- Relationship between virus titer in serum and transmission of ZIKV to bodies of *Ae. albopictus* from cynomolgus macaques (light blue) or squirrel monkeys (dark blue). Points show raw data, with point size proportional to the number of mosquitoes tested in the batch (one day on one monkey). B-F - Relationships between virus titer in serum and transmission of ZIKV to bodies of *Ae. albopictus* from both NHP species (blue curve, repeated in each panel) or transmission to bodies of *Ae. aegypti* of DENV serotype 1-4 from humans (B-F). DENV curves fitted to data from Nguyen et al. 2013[10] in sub-panels B,C,E,F, and data from Long et al. 2019[13] in sub-panel D. Infectious dose 50 (ID_{50}), and confidence interval (CI), provided for each designated virus and mosquito species in $\log_{10}$ PFU/ml. Source data are provided as a Source Data file.

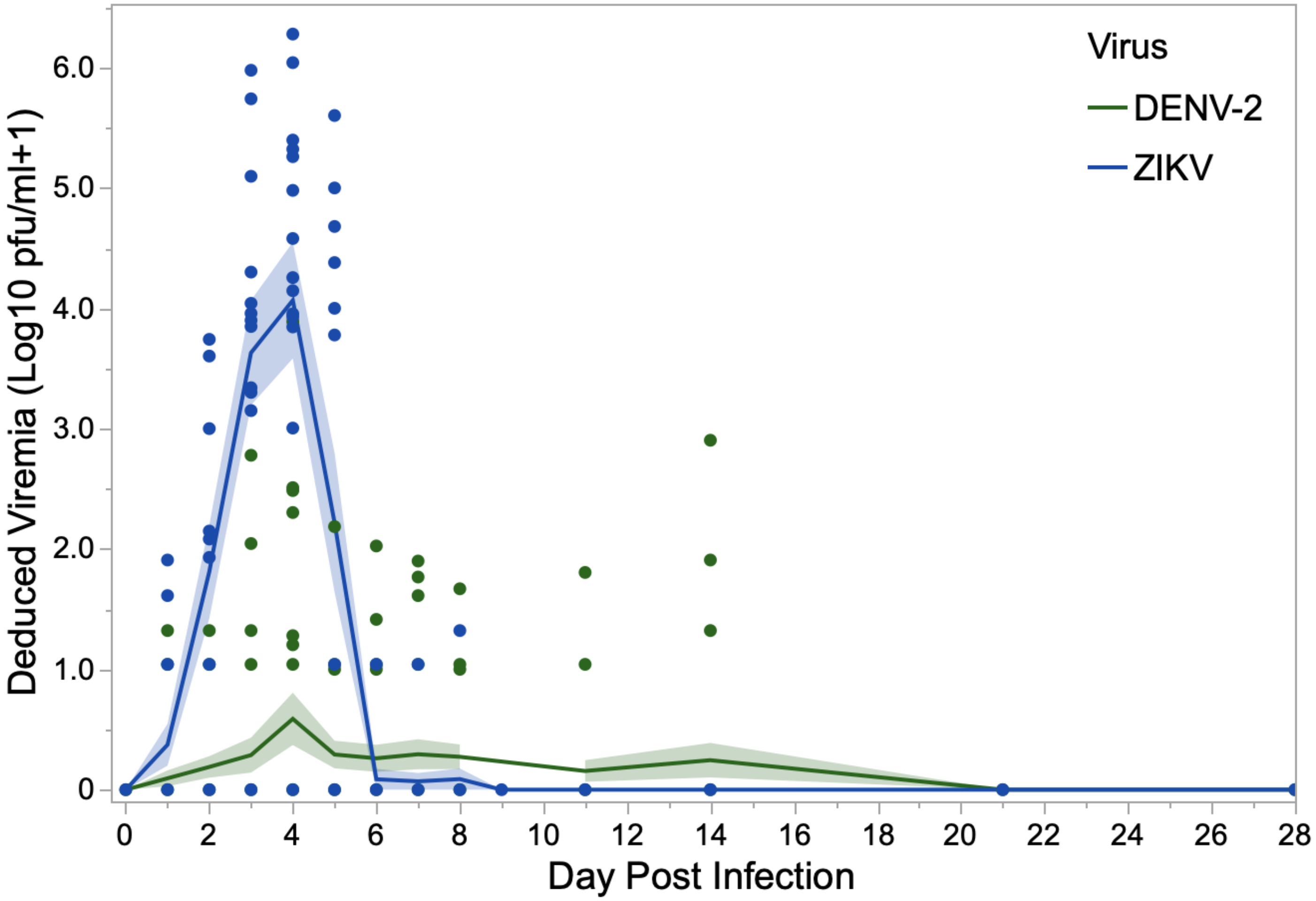

Figure S.5: Deduced viremia from macaques and squirrel monkeys infected with DENV-2 or ZIKV at each dpi. Lines show mean and shaded bands show standard errors. See text above for how these viremia values sometimes differ from the initial result of the assays. Source data are provided as a Source Data file.

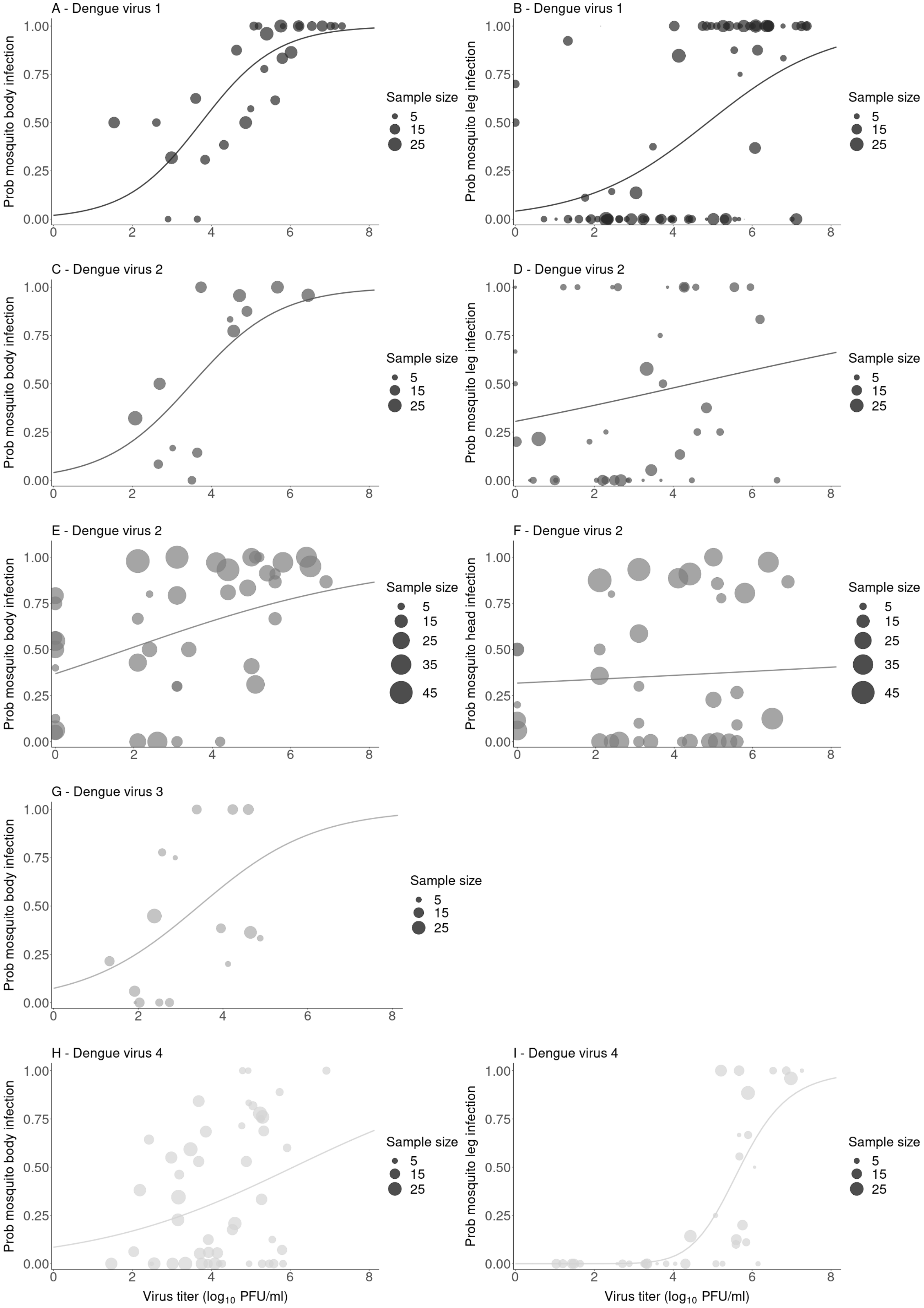

Figure S.6: (Caption next page)

Figure S.6: (Previous page.) Dose-response curves fitted to mosquito body infection data (left column) and mosquito leg infection data (right column) for DENV. Data from the literature : data from Nguyen et al. 2013[10] in sub-panels A,C,G,H ; data from Duong et al. 2015[12] in sub-panels B,D,I ; data from Long et al. 2019[13] in sub-panels E,F. Note that as we did not know the value of the limit of detection (LOD) for dengue studies, points with zero viremia and transmission to mosquitoes have been used for fitting, whereas for Zika virus those points were assigned a viremia of 10 PFU (half the LOD of our assays). Y-axis in F is probability of mosquito head infection, another marker of disseminated infection. Viral loads in E and F are in FFU/ml, which we considered equivalent to PFU/ml. Source data are provided as a Source Data file.

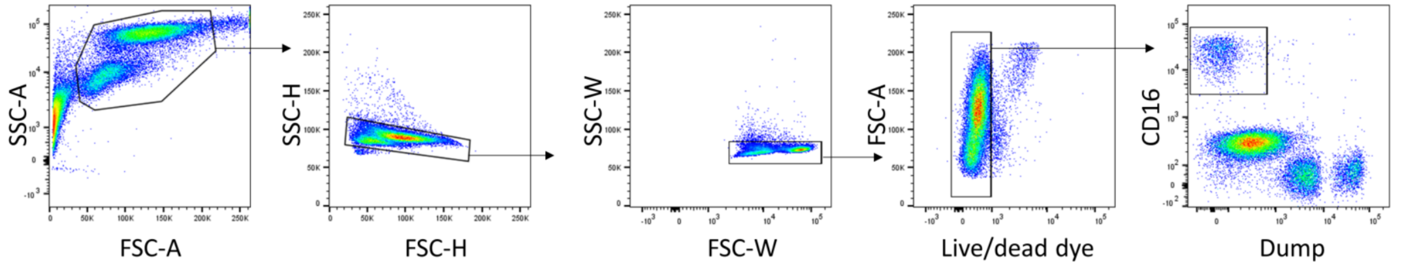

Figure S.7: Flow cytometry gating strategy. Leukocytes were initially gated based on forward scatter area (FSC-A) and side scatter area (SSC-A) parameters. Subsequently, single cells were identified using FSC-H and SSC-H, as well as FSC-W and SSC-W gating. To exclude dead cells, a Live/Dead cell dye was utilized. NK cells were specifically identified within the leukocyte population by gating on CD16^{+} Dump channel- (CD3^{+}/CD14^{+}/CD20^{+}) subsets.

References

1. Fortman, J., Hewett, T., Bennett, B. Important biologic features. Chapter 1. in *The laboratory nonhuman primate* (eds Fortman, J., Hewett, T., Bennett, B.) pg 17 (Boca Raton, FL: CRC, 2002)

2. Hrapkiewicz K and Medina L. Non-human primates. in *Clinical Laboratory Animal: An Introduction* pg 287 (Blackwell Publishing, Ames, Iowa, USA, 2007)

3. *The Merck Veterinary Manual.* (Whitehouse Station, NJ :Merck & Co., Inc.)

4. Brady, A. G. Research Techniques for the Squirrel Monkey (Saimiri sp.). *ILAR Journal*, **41**, 1 (2000)

5. Marquardt, N. et al. The human NK cell repsonse to Yellow Fever virus 17D is primarily governed by NK cell differentiation independently of NK cell education. *The Journal of Immunology* **195**, 3262–3272 (2015).

6. Björkström, N. K., Strunz, B. & Ljunggren, H.-G. Natural killer cells in antiviral immunity. *Nat Rev Immunol* **22**, 112–123 (2022).

7. Burnham, K. P., Anderson, D. R. *Model Selection and Multimodel Inference : a Practical Information-theoretic Approach.* Second Edition. (Springer Science+Business Media New York, 2002)

8. Ferguson, N. M. et al. Modeling the impact on virus transmission of Wolbachia-mediated blocking of dengue virus infection of Aedes aegypti. *Sci. Transl. Med.* **7**, 279-279ra37 (2015).

9. Somvanshi, P. R., Venkatesh, K.V. Hill Equation. in *Encyclopedia of Systems Biology* (eds Dubitzky, W., Wolkenhauer, O., Cho, K. H., Yokota, H.) 892-895 (Springer New York, 2013)

10. Nguyen, N. M. et al. Host and viral features of human dengue cases shape the population of infected and infectious Aedes aegypti mosquitoes. *Proc. Natl. Acad. Sci. U.S.A.* **110**, 9072–9077 (2013).

11. ten Bosch, Q. A. et al. Contributions from the silent majority dominate dengue virus transmission. *PLOS Pathogens* **14**, e1006965 (2018).

12. Duong, V. et al. Asymptomatic humans transmit dengue virus to mosquitoes. *Proc. Natl. Acad. Sci. U.S.A.* **112**, 14688–14693 (2015).

13. Long, K. C. et al. Feasibility of feeding Aedes aegypti mosquitoes on dengue virus-infected human volunteers for vector competence studies in Iquitos, Peru. *PLoS Negl Trop Dis* **13**, e0007116 (2019).

14. Blaney, J. E., Matro, J. M., Murphy, B. R. & Whitehead, S. S. Recombinant, live-attenuated tetravalent dengue virus vaccine formulations induce a balanced, broad, and protective neutralizing antibody response against each of the four serotypes in rhesus monkeys. *J. Virol.* **79**, 13 (2005).
