# Supplementary material for: Trade-offs shaping transmission of sylvatic dengue and Zika viruses in monkey hosts
Source: Nat Commun. 2024 Mar 27;15:2682. doi: 10.1038/s41467-024-46810-x (PMC10973334; doi:10.1038/s41467-024-46810-x)
Supplement: Supplementary file 3 — Description of Additional Supplementary Files [file 41467_2024_46810_MOESM3_ESM.pdf]

Description of Additional Supplementary Files

Supplementary Data 1: Raw data for cynomolgus macaques (Macaca fascicularis) infected with sylvatic dengue virus serotype 2

Supplementary Data 2: Raw data for squirrel monkeys (Saimiri boliviensis) infected with sylvatic dengue virus serotype 2

Supplementary Data 3: Raw data for cynomolgus macaques (Macaca fascicularis) infected with sylvatic Zika virus

Supplementary Data 4: Raw data for squirrel monkeys (Saimiri boliviensis) infected with sylvatic Zika virus

Supplementary Data 5. 50% Plaque Reduction Neutralization Titer Values (PRNT50, inverse of dilution) for cynomolgus macaques or squirrel monkeys against designated viruses prior to and 28 days after infection with designated virus (see text for information on virus strains).

Supplementary Data 6. Virus titer for each mosquito with any tissue that was positive for infection
