# Supplementary material for: Trade-offs shaping transmission of sylvatic dengue and Zika viruses in monkey hosts
Source: Nat Commun. 2024 Mar 27;15:2682. doi: 10.1038/s41467-024-46810-x (PMC10973334; doi:10.1038/s41467-024-46810-x)
Supplement: Supplementary file 10 — Reporting Summary [file 41467_2024_46810_MOESM10_ESM.pdf]

nature portfolio

Corresponding author(s): Kathryn A. Hanley

Last updated by author(s): 02/24/2024

Reporting Summary

Nature Portfolio wishes to improve the reproducibility of the work that we publish. This form provides structure for consistency and transparency in reporting. For further information on Nature Portfolio policies, see our Editorial Policies and the Editorial Policy Checklist.

Statistics

For all statistical analyses, confirm that the following items are present in the figure legend, table legend, main text, or Methods section.

n/a	Confirmed	
☐	☒	The exact sample size (*n*) for each experimental group/condition, given as a discrete number and unit of measurement
☐	☒	A statement on whether measurements were taken from distinct samples or whether the same sample was measured repeatedly
☐	☒	The statistical test(s) used AND whether they are one- or two-sided<br>*Only common tests should be described solely by name; describe more complex techniques in the Methods section.*
☐	☒	A description of all covariates tested
☐	☒	A description of any assumptions or corrections, such as tests of normality and adjustment for multiple comparisons
☐	☒	A full description of the statistical parameters including central tendency (e.g. means) or other basic estimates (e.g. regression coefficient) AND variation (e.g. standard deviation) or associated estimates of uncertainty (e.g. confidence intervals)
☐	☒	For null hypothesis testing, the test statistic (e.g. *F*, *t*, *r*) with confidence intervals, effect sizes, degrees of freedom and *P* value noted<br>*Give P values as exact values whenever suitable.*
☒	☐	For Bayesian analysis, information on the choice of priors and Markov chain Monte Carlo settings
☒	☐	For hierarchical and complex designs, identification of the appropriate level for tests and full reporting of outcomes
☒	☐	Estimates of effect sizes (e.g. Cohen's *d*, Pearson's *r*), indicating how they were calculated

Our web collection on statistics for biologists contains articles on many of the points above.

Software and code

Policy information about availability of computer code

Data collection	Flow cytometry data was acquired using a BD Fortessa running FACSDiva 8.0.1 for data acquisition
Data analysis	Flow cytometry data was analyzed utilizing FLOWJO_v10.7.1 (FlowJo, Ashland, OR, USA).; code for all other analyses found at https://github.com/helenececilia/hanley_2023_sylvatic_DENV_ZIKV_trade_offs. R version 4.3.1 was used, and analyses relied on packages glmmTMB 1.1.7, DHARMa 0.4.6, multcomp 1.4-25, car 3.1-2, bbmle 1.0-25, mgcv 1.9-0, emdbook 1.3.13, tmvtnorm 1.5.

For manuscripts utilizing custom algorithms or software that are central to the research but not yet described in published literature, software must be made available to editors and reviewers. We strongly encourage code deposition in a community repository (e.g. GitHub). See the Nature Portfolio guidelines for submitting code & software for further information.

Data

Policy information about availability of data

All manuscripts must include a data availability statement. This statement should provide the following information, where applicable:

- Accession codes, unique identifiers, or web links for publicly available datasets
- A description of any restrictions on data availability
- For clinical datasets or third party data, please ensure that the statement adheres to our policy

All raw data from macaques infected with DENV-2 and ZIKV and squirrel monkeys infected with DENV-2 or ZIKV, save for temperature and PRNT values against non-

infecting viruses, are provided in Supplemental Tables S.1, S.2,S.3 and S.4. For additional data, see https://github.com/helenececilia/hanley_2023_sylvatic_DENV_ZIKV_trade_offs

Research involving human participants, their data, or biological material

Policy information about studies with human participants or human data. See also policy information about sex, gender (identity/presentation), and sexual orientation and race, ethnicity and racism.

Reporting on sex and gender	N/A
Reporting on race, ethnicity, or other socially relevant groupings	N/A
Population characteristics	N/A
Recruitment	N/A
Ethics oversight	N/A

Note that full information on the approval of the study protocol must also be provided in the manuscript.

Field-specific reporting

Please select the one below that is the best fit for your research. If you are not sure, read the appropriate sections before making your selection.

☒ Life sciences ☐ Behavioural & social sciences ☐ Ecological, evolutionary & environmental sciences

For a reference copy of the document with all sections, see nature.com/documents/nr-reporting-summary-flat.pdf

Life sciences study design

All studies must disclose on these points even when the disclosure is negative.

Sample size	To test a priori predictions within and between each species-virus combination, we used standard parametric tests or generalized linear models, as appropriate. A priori power analyses were conducted to assess our ability to detect changed in peak titer and duration of titer between low and high dose DENV-2 infections in macaques, resulting in a sample size of 10 in the infected group. As one macaque was transferred to the control group, and as analyses were primarily focused on NK cells and neutralizing antibody titers due to the paucity of viremia, we conducted a post-hoc test of power, and found that, with the effect sizes and standard deviations reported, we had approximately 75% power to detect a significant difference in comparisons among 2 or 3 groups and lower power of approxi-mately 20% for linear regressions. Sample sizes were smaller for infected squirrel monkeys (N = 8 per virus) and power was concomitantly lower. For models testing interactions, if the interactions term was non-significant with an effect size $\eta 2 < 0.01$, we relied on a type II anova for the significance of main effects, following recommendations by Smith and Cribbie 59. Sample size of ZIKV-infected macaques was N = 3, precluding most individual statistical analyses.
Data exclusions	For analyses of the magnitude and duration of viremia, we chose to exclude 4683, the animal eu-thanized at 13 dpi (Supplemental Text S.6), as we did not know whether pre-existing health prob-lems may ultimately have led to euthanasia and may have impacted viral replication or immune pa-rameters; however, we note throughout when inclusion of this animal altered determination of significance of comparisons.
Replication	Within-host dynamics and transmission of sylvatic DENV-2 were tested in two separate hosts (macaques and squirrel monkeys). While this does not constitute replication a priori, since we did expect patterns to differ between the two hosts, instead we found remarkable similarity between them. We note that, particularly for non-human primates, there is a tension between minimizing animal use and reproducing experiments.
Randomization	Monkeys were stratified by sex and then randomized into treatments using a random number generator. If this randomization resulted in a significant difference in the mean weight of monkeys in each treatment, then the process was repeated until weights were not significantly different.
Blinding	Animal care staff were blinded to treatment, as was the pathologist; the remaining data was quantitative (titers, cytokine concentrations, flow cytometry data) and did not require blinding.

Reporting for specific materials, systems and methods

We require information from authors about some types of materials, experimental systems and methods used in many studies. Here, indicate whether each material, system or method listed is relevant to your study. If you are not sure if a list item applies to your research, read the appropriate section before selecting a response.

Materials & experimental systems

n/a	Involved in the study
☐	☒ Antibodies
☐	☒ Eukaryotic cell lines
☒	☐ Palaeontology and archaeology
☐	☒ Animals and other organisms
☒	☐ Clinical data
☒	☐ Dual use research of concern
☒	☐ Plants

Methods

n/a	Involved in the study
☒	☐ ChIP-seq
☐	☒ Flow cytometry
☒	☐ MRI-based neuroimaging

Antibodies

Antibodies used

For virus detection, mouse hyperimmune serum against DENV-2 NGC (MIAF T-35432, generated 12/10/2014) or mouse hyperimmune serum against ZIKV strain MR-766 (MIAF T-36846 generated 07/19/2017) or pan-flavivirus monoclonal antibody 4G2 were used. All three antibodies were used at a 1:2000 dilution; MIAF T-35432 and MIAF T-36846 mouse hyperimmune antibodies were obtained from the World Reference Center on Emerging Viruses and Arboviruses (WRCEVA) at UTMB University of Texas Medical Branch while 4G2 was obtained from VWR (Radnor, PA, catalog number 76005-718). The plates were then washed with PBS, followed by incubation with a goat anti-mouse secondary antibody conjugated to horseradish peroxidase (KPL, Gaithersburg, MD, USA, catalog number 5220-0341) diluted 1:2000 in blocking solution.

For NK cell quantification, all antibodies were purchased from BD Biosciences and used at the manufacturer recommended dilution. Cells were stained with mouse anti-human CD20 (clone 2H7, Alexa-700, catalog number 560631, lot number 0209371, 1:16 dilution), mouse anti-human CD3 (clone SP34-2, Alexa-700, catalog number catalog number 557917, lot number 9185577, 1:16 dilution), mouse anti-human CD14 (clone M5E2, Alexa-700, catalog number 557923, lot number 0023216, 1:8 dilution), and mouse anti-human CD16 (clone 3G8, PE, catalog number 556619, lot number 9107538, 1:18 dilution).

Validation

Both mouse hyperimmune sera have been validated in our laboratories and show to be highly sensitive and moderately specific for DENV and ZIKV, respectively. Mouse hyperimmune sera have been used extensively in vaccine development studies for both viruses. Regarding MIAF T-35432:This antibody has been used in a large body of our work on flaviviruses, such as Vasilakis et al. Virology 2013 (doi: 10.1016/j.virol.2006.08.049). Regarding MIAF T-36846: This antibody has been used in a large body of our work on flaviviruses, such as Vasilakis et al. Virology 2013 (doi: 10.1016/j.virol.2006.08.049).

Regarding 4G2, This antibody has been used in a large body of our work on flaviviruses, such as Azar et al. Viruses 2022 (doi: 10.3390/v14040665), Hanley et al. Viruses 2019 (doi: 10.3390/v11111072), Vasilakis et al. Virology 2013 (doi: 10.1016/j.virol.2006.08.049). The VWR website states: This antibody binds to flavivirus group antigen, protein E. It can be used as an anti-Dengue virus antibody, anti-West Nile virus antibody, anti-Japanese Encephalitis or anti-Zika Virus antibody (Aubry et al. 2016) to identify cells infected with these flaviviridae. It binds to the fusion loop at the extremity of domain II of E protein from all four serotypes and prevents syncytia formation. The epitope is highly conserved amongst flaviviridae and has been functionally analyzed in detail by Crill and Chang 2004. Previous studies have used acetone- (Henchal et al. 1982) or methanol-fixed slides (Moreland & Tay, 2010).

Regarding the secondary goat-anti-mouse antibody used for virus detection, this antibody has been used in a large body of our work, e.g. Chen et al. Cell Host Microbe 2022 (doi: 10.1016/j.chom.2021.09.006) as well as in vaccine development studies.

Regarding mouse anti-human CD20, Ram et al. Frontiers in Immunology 2020 have recently used this antibody clone for profiling of NK cells in rhesus macaques (DOI: 10.3389/fimmu.2020.01676). The BD biosciences website states: The 2H7 monoclonal antibody specifically binds to CD20, encoded by the MS4A1 (Membrane-spanning 4-domains, subfamily A, member 1) gene. CD20 is a 33-37 kDa, unglycosylated four-transmembrane phosphoprotein. CD20 is expressed on pre-B-cells, resting and activated B cells, and follicular dendritic cells, but not plasma cells. Low level CD20 expression is observed on a small subset of normal circulating T lymphocytes. The CD20 molecule is involved in the regulation of B-cell activation. This clone also cross-reacts with a subset of peripheral blood lymphocytes, but not monocytes nor granulocytes, of baboon and both rhesus and cynomolgus macaque monkeys. The distribution on lymphocytes is similar to that seen with normal human donor lymphocytes, namely bright staining on B lymphocytes and weak reactivity on a small subset of CD3-positive T lymphocytes.

Regarding mouse anti-human CD3, Ram et al. Frontiers in Immunology 2020 have recently used this antibody clone for profiling of NK cells in rhesus macaques (DOI: 10.3389/fimmu.2020.01676). The BD biosciences website states: Clone SP34-2 is a mouse IgG1 isotype monoclonal antibody, descendant of SP34 (mouse IgG3), with the same specificity and reactivity pattern as the parent clone. It cross-reacts with a major subset of peripheral blood lymphocytes, but not monocytes or granulocytes, of baboon, and rhesus, cynomolgus, and pigtail macaque monkeys. The distribution on lymphocytes is similar to that observed with normal human donor lymphocytes with the majority of CD3-positive cells being negative when dual stained with antibodies to B or NK cells markers. SP34-2 is also capable of inducing cell proliferation on both human and non-human primate PBMC.

Regarding mouse anti-human CD14, Ram et al. Frontiers in Immunology 2020 have recently used this antibody clone for profiling of NK cells in rhesus macaques (DOI: 10.3389/fimmu.2020.01676). The BD biosciences website states: The M5E2 monoclonal antibody specifically binds to CD14, a 53–55 kDa glycosylphosphatidylinositol (GPI)-anchored single chain glycoprotein expressed at high levels on monocytes. Additionally, the anti-CD14 antibody reacts with interfollicular macrophages, reticular dendritic cells, and some Langerhans cells. CD14 has been identified as a high affinity cell-surface receptor for complexes of lipopolysaccharide (LPS) and serum LPS-binding protein, LPB.

Regarding mouse anti-human CD16, Choi et al. Immunology 2008 have used this antibody clone for in vivo depletion of NK cells in

rhesus macaques (doi: 10.1111/j.1365-2567.2007.02757.x). The BD biosciences website states: The 3G8 monoclonal antibody specifically recognizes CD16a and CD16b, low affinity receptors for the Fc region of IgG. CD16a is ~50-65 kDa type I transmembrane glycoprotein that is encoded by FCGR3A (Fc fragment of IgG receptor IIIa) which belongs to the immunoglobulin superfamily. CD16a is also known as Fc-gamma RIII-alpha (Fc-gamma RIIIa or FcγRIIIA) or FcRIIIa and is expressed on natural killer cells, activated monocytes, macrophages, γδ T cells, immature thymocytes, and mast cells. CD16a binds immune-complexed or aggregated IgG and associates with CD247/TCRζ in NK cells and FcεRIγ chains in phagocytes and mast cells to transduce intracellular signals. CD16a functions in antibody-dependent cellular cytotoxicity (ADCC) and other antibody-dependent responses including phagocytosis, cytokine production or mediator release. CD16b is a ~48 kDa glycophosyl-phosphatidylinositol (GPI)-linked form that is encoded by FCGR3B (Fc fragment of IgG receptor IIIb). CD16b is also known as Fc-gamma RIII-beta (Fc-gamma RIIIb or FcγRIIIB) or FcRIIIb and is expressed on neutrophils and activated eosinophils. The extracellular region of CD16b is highly homologous to CD16a. CD16b also serves as a receptor for the Fc region of IgG and can bind immune-complexed or aggregated IgG and may be involved in neutrophil adhesion. The 3G8 antibody also crossreacts with a subset of peripheral blood lymphocytes and monocytes, but not granulocytes, of baboon, rhesus, and cynomolgus monkeys. Multicolor analysis reveals that the distribution on lymphocytes is similar to that found in human studies with the majority of CD16-positive lymphocytes being both CD3 and CD20 negative.

Eukaryotic cell lines

Policy information about cell lines and Sex and Gender in Research

Cell line source(s)	African green monkey kidney cells (Vero) and larval Ae. albopictus cells (C6/36) were purchased from the American Type Culture Collection (ATCC, Bethesda, MD, USA).
Authentication	Cell lines were authenticated in both laboratories annually via PCR
Mycoplasma contamination	Cell lines were tested annually for mycoplasma and were negative
Commonly misidentified lines (See ICLAC register)	No commonly misidentified cell lines were used in this research.

Animals and other research organisms

Policy information about studies involving animals; ARRIVE guidelines recommended for reporting animal research, and Sex and Gender in Research

Laboratory animals	Mauritius origin adolescent Macaca fascicularis (between 2.4 to 5.4 years of age) weighing between 2.75-5.4 kg were purchased from Worldwide Primates, Inc (Miami, FL, USA). Macaques were single housed in open metal caging that allowed for visual but not physical contact with other animals in the room. Standard primate chow was provided twice daily, with enrichment in the form of fruits and vegetables added once daily. All animals received a minimum of twice daily health checks.<br><br>Saimiri boliviensis boliviensis (Black capped squirrel monkeys) over an adolescent to adult age range (between 4 to 14 years of age) were purchased from the MD Anderson Center (Bastrop, TX, USA). Squirrel monkeys were pair housed in open metal caging that allowed for visual but not physical contact with animals in other cages. Standard primate chow was provided twice daily, with enrichment in the form of fruits and vegetables added once daily. All animals received a minimum of twice daily health checks.<br><br>Aedes albopictus were field collected in Galveston Texas in the summer of 2018 and utilized to establish a continuous colony. All mosquitoes utilized over the course of these experiments were derived from this original colony. In Experiment 1, Ae. albopictus Galveston F12 were used, while in Experiment 2, Ae. albopictus Galveston F14 were used. Mosquito colonies were maintained under controlled insectary conditions. (28 ± 1 °C with 80 ± 10% RH and a 16:8 light:dark cycle). Mosquito experiments used female adults 4-7 days post-eclosion.
Wild animals	No wild animals were used in this study
Reporting on sex	Both sexes of both cynomolgus macaques and squirrel monkeys were used in both treatments. This study was not designed or powered to test the effect of sex.
Field-collected samples	Aedes albopictus were field collected in Galveston Texas in the summer of 2018 and utilized to establish a continuous colony. All mosquitoes utilized over the course of these experiments were derived from this original colony. Mosquito colonies were maintained under controlled insectary conditions (28 ± 1 °C with 80 ± 10% RH and a 16:8 light:dark cycle). All engorged mosquitoes were homogenized and used to assay virus titer; all unengorged mosquitoes were killed by freezing and autoclaved for disposal.
Ethics oversight	All procedures conducted on non-human primates were approved via UTMB Institutional Animal Care and Use Committee (IACUC) protocol 1912100

Note that full information on the approval of the study protocol must also be provided in the manuscript.

Flow Cytometry

Plots

Confirm that:

☒ The axis labels state the marker and fluorochrome used (e.g. CD4-FITC).

☒ The axis scales are clearly visible. Include numbers along axes only for bottom left plot of group (a 'group' is an analysis of identical markers).

☒ All plots are contour plots with outliers or pseudocolor plots.

☒ A numerical value for number of cells or percentage (with statistics) is provided.

Methodology

Sample preparation	Lithium heparin microtainer tubes containing 0.2 – 0.3 ml blood were taken into a biosafety cabinet and 200 µL of heparinized whole blood from each NHP was added to an individually labelled round-bottomed polystyrene tube (Corning/ Falcon, Corning, NY, USA). Nonspecific antigen binding to Fc receptors was inhibited via 10 minutes of blocking with Human TruStain FcX (BioLegend, San Diego, CA, USA). Cells were subsequently stained with mouse anti-human CD20 (Alexa-700), mouse anti-human CD3 (Alexa-700), mouse anti-human CD14 (Alexa-700), mouse anti-human CD16 (PE) (all antibodies purchased from BD Biosciences), and Live/Dead Fixable Blue (Invitro-gen, Waltham, MA, USA) in a volume of 100 µL for 1 hour in the dark with gentle agitation every 15 minutes. Subsequently, erythrocytes were lysed with BD FACS Lysing Solution (BD Bioscienc-es) diluted in molecular biology grade water (Corning, Corning, NY, USA) for 12 minutes followed by immediate centrifugation at 500g for 5 minutes at room temperature. Supernatant was immediate-ly aspirated and the cellular pellet was reconstituted in FACS Buffer (0.2% bovine serum albumin, 2mM EDTA in phosphate buffered saline). Samples were centrifuged at 350g for 5 minutes at room temperature. FACS buffer was removed and pellets were fixed in freshly prepared 2% methanol free formaldehyde (diluted from 16% Ultra Pure EM grade formaldehyde (Polysciences, Warrington, PA, USA) for a minimum of 1 hour prior to analysis. Analyses were conducted using a BD LSRFortessa Cell Analyzer (BD, Franklin Lakes, NJ, USA), and 105 total cells were analyzed. Natural killer cells were defined as the live cell population that were CD16+ and CD3-CD14-CD20-.
Instrument	Flow cytometry data was acquired using a BD Fortessa running FACSDiva 8.0.1 for data acquisition.
Software	Flow cytometry data was analyzed utilizing FLOWJO_v10.7.1 (FlowJo, Ashland, OR, USA).
Cell population abundance	The fraction of NK cells within total PBMCs is reported in the manuscript and in Supplemental Tables S.1, S.2 and S.3. These are the only relevant cell fraction for this study.
Gating strategy	Leukocytes were initially gated based on forward scatter area (FSC-A) and side scatter area (SSC-A) parameters. Subsequently, single cells were identified using FSC-H and SSC-H, as well as FSC-W and SSC-W gating. To exclude dead cells, a Live/Dead cell dye was utilized. NK cells were specifically identified within the leukocyte population by gating on CD16+ Dump channel- (CD3+/CD14+/CD20+) subsets.

☒ Tick this box to confirm that a figure exemplifying the gating strategy is provided in the Supplementary Information.
